# Supplementary material for: Systematic profiling of ale yeast protein dynamics across fermentation and repitching
Source: G3 (Bethesda). 2023 Dec 22;14(3):jkad293. doi: 10.1093/g3journal/jkad293 (PMC10917522; doi:10.1093/g3journal/jkad293)
Supplement: jkad293_Supplementary_Data [file jkad293_supplementary_data.zip › Supplemental_Material_G3-2023-404592.docx]

**Supplementary Figures**

**Supplementary Figure 1. Genomic characteristics of Wyeast 3068.** A) Mean sequencing coverage across the Wyeast 3068 genome. Each dot represents the mean coverage (y-axis) of a particular locus along the genome position (x-axis). B) Local copy number as an average read depth over 1000bp windows normalized to the known ploidy of 4n. C) Allele frequencies (y-axis) of called variants. (Note that genome positions in A, B, and C have been concatenated for whole-genome display). D) Comparison between read depth (x-axis) at each gene and level of the corresponding protein abundance (y-axis). Adjusted R^2^=0.00008246, p≤0.2703. E) Comparison between average read depth/chromosome (x-axis) and average LFQ abundance of corresponding protein by chromosome (y-axis), adjusted R^2^=-0.04194, p≤0.5392.

**Supplementary Figure 2. Fermentation proteomics summary statistics.** A) Scatterplots depicting the correlation between replicates 1 and 2 for each fermentation time point assayed. Axes indicate the log-transformed observed LFQ intensity calculated using MaxQuant. B) Pairwise similarity matrix showing the extent of correlation across all sampled replicates and time points in the dataset. C) Barplots summarizing the number of proteins detected in each time point colored by detection in both (orange), one (yellow), or undetected (blue). D) Dotplots with top enriched cellular component GO terms for proteins detected only across batches 1 and 15 and not in the conditioning tank. Terms were filtered for at a 5% FDR threshold and Benjamini-Hochberg adjusted p-value of < 0.05. E) Boxplots comparing the distributions of protein molecules per cell as reported previously (Ho *et al.* 2018).

**Supplementary Figure 3. Subcellular proteome analysis.** A) Barplots representing the numbers of yeast proteins (x-axis) annotated by subcellular/organellar location (y-axis). Data curated from the Yeast GFP Fusion Localization database (Huh *et al.* 2003) and B) Fraction of proteins in each subcellular/organellar location detected in at least one time point across the brewing time course. C) Densities of all Pearson correlation values calculated across all pairs of proteins across each annotated subcellular location and organelles. D) Histogram of the distribution of yeast protein complex sizes. Data obtained from Yeast complexome (Meldal *et al.* 2019). E) Density plot showing the fraction of yeast protein complexes detected in the fermentation time course. Protein complex data curated from the EBI Complexome database (Meldal *et al.* 2019).

**Supplementary Figure 4. Uncovering protein complex regulation across brewing.** A) Left, Pairwise scatterplots of abundance comparing protein pairs Fas1-Fas2, and Fas1-Erg6 All by all pairwise protein Pearson correlation matrix where rows and columns represent all 2572 well-detected proteins across the fermentation dataset. B) Density plot of correlation between known interacting and non-interacting protein pairs (Interaction data curated from EBI complexome (Meldal *et al.* 2019)). C) Cytosolic ribosomal subunits tend to be more correlated than their mitochondrial counterparts. Density plots of pairwise correlation among protein complex members involved in: D) Chromatin remodeling, E) Transcription, F) Vesicle transport, and G) Protein regulation.

**Supplementary Figure 5. Comparing differentially expressed proteins across shared time points between batches 1 and 15.** A) Scatter plots of summed LFQ intensity across replicates comparing time points sampled in both Batch 1 (x-axis) and Batch 15 (y-axis) brewing cycles. B) Differentially expressed proteins across matched time points (0h, 24h, 48h, 72h, 96h, and 24PC) between Batches 1 and 15 depicted using volcano plots with log_2_ fold change (x-axis) and Benjamini-Hochberg adjusted p-value (y-axis). C) Dotplots with the top enriched GO terms for the matched starting time point across Batch 1 and Batch 15. D) Dotplots with top enriched GO terms across Batch 15 3h and Batch 1 6h time points. GO term analysis was performed on the biological function terms with a 5% FDR threshold and filtering terms to an adjusted p-value (Benjamini-Hochberg correction) of <0.05. Sizes of dots correspond to the ratio genes detected to the total genes annotated for a particular GO term.

**Supplementary Figure 6. Cataloging abundance changes in metabolic pathways.** A) Pairwise Pearson correlation between proteins involved in the glycolysis and tricarboxylic acid (TCA) cycle pathways. B) LFQ protein abundance (y-axis) as a function of time (x-axis) for enzymes involved in pyruvate metabolism. Time points colored by batches 1 (red) and 15 (blue) C) Steps in pyruvate metabolism in yeast. D) Changes in fatty acid oxidation and E) very long chain fatty acid synthesis enzyme abundances, over both batches and during final conditioning, as log_2_ of row mean normalized abundance.

**Supplementary Tables**

**Supplementary Table 1. List of deletions and affected genes with chromosomal coordinates to the nearest 100bp.**

**Supplementary Table 2. List of time points sampled with mass spectrometry.**

**Supplementary Table 3. Sum of LFQ values for each detected protein matched with sequencing coverage of corresponding gene.**

**Supplementary Table 4. Correlation between detected protein levels and sequencing coverage.**

**Supplementary Table 5. Protein detection statistics by time point.**

**Supplementary Table 6. Log_2_ fold change normalized to the mean abundance values for all detected proteins across both Batch 1 and Batch 15 time points.**

**Supplementary Table 7. Clustering of proteins that changed at least two-fold over the mean in any time point.**

**Supplementary Table 8. List of GO terms enriched in each cluster.**

**Supplementary Table 9. Number of proteins identified from different subcellular locations.**

**Supplementary Table 10. Yeast protein complex detection statistics across time course dataset.**

**Supplementary Table 11. Summary of differentially expressed proteins across time points.**

**Supplementary Table 12. List of GO terms (biological process) for differentially expressed proteins across select time points.**

**Supplementary Table 13. Top 100 metabolic pathways from SGD Yeast Pathways.**
