# Supplementary figures and images for: Systematic profiling of ale yeast protein dynamics across fermentation and repitching
Source: G3 (Bethesda). 2023 Dec 22;14(3):jkad293. doi: 10.1093/g3journal/jkad293 (PMC10917522; doi:10.1093/g3journal/jkad293)

A

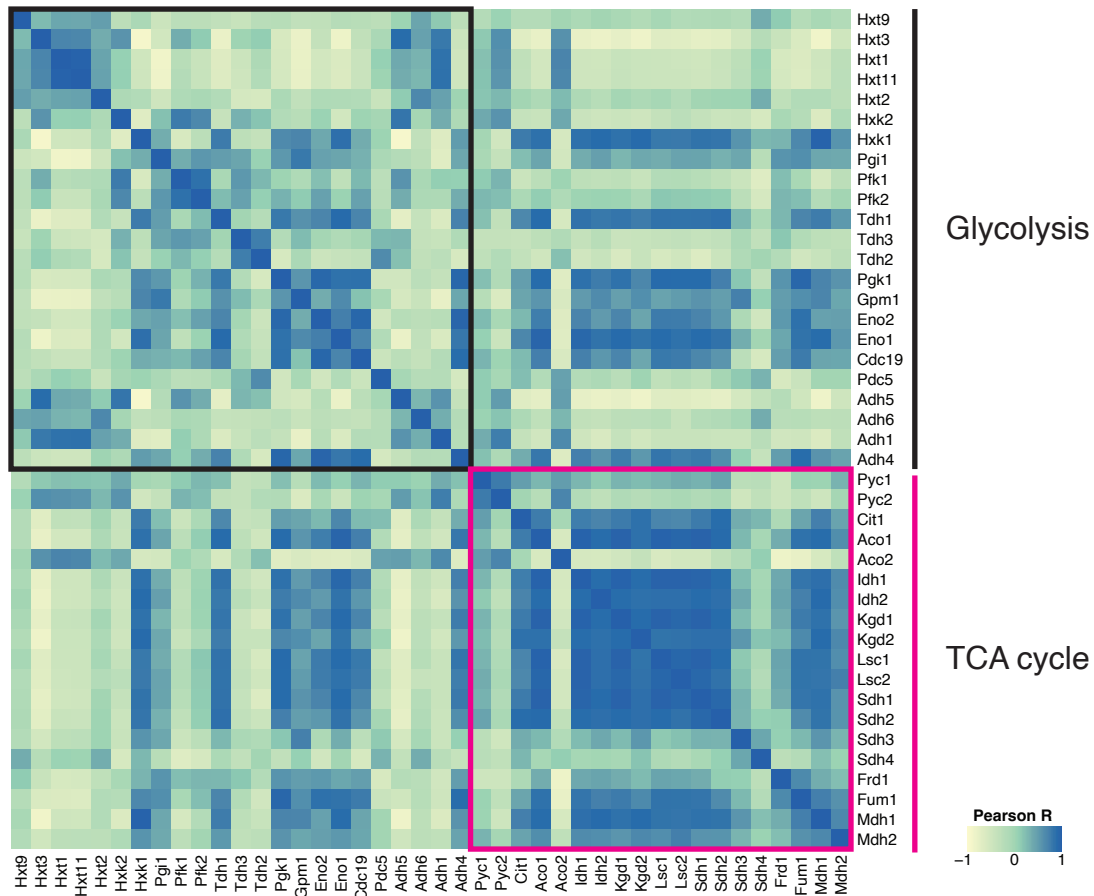

B

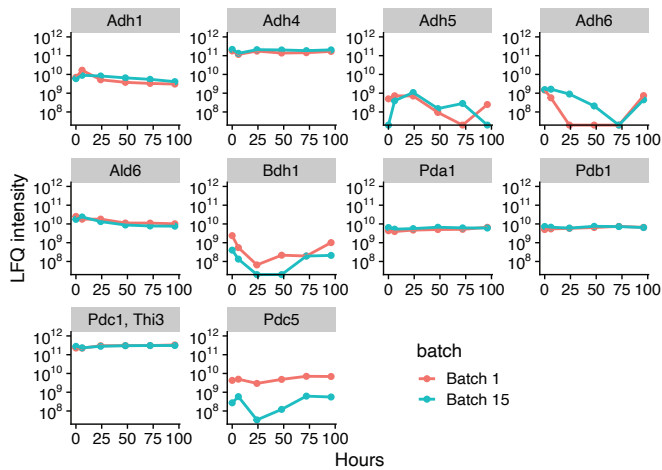

C

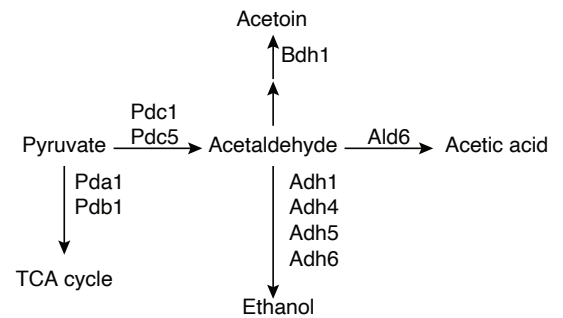

D

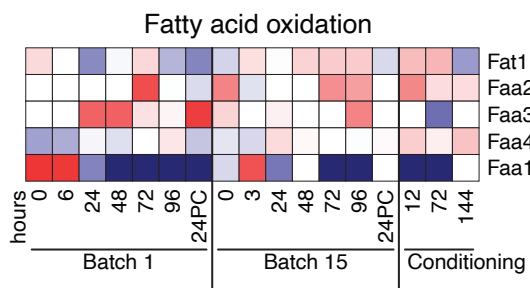

E

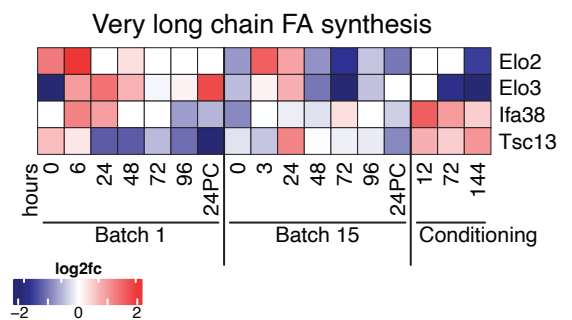

Supplementary Figure 6

Supplement: jkad293_Supplementary_Data [file jkad293_supplementary_data.zip › Supplementary_Figure_6_G3-2023-404592.pdf]

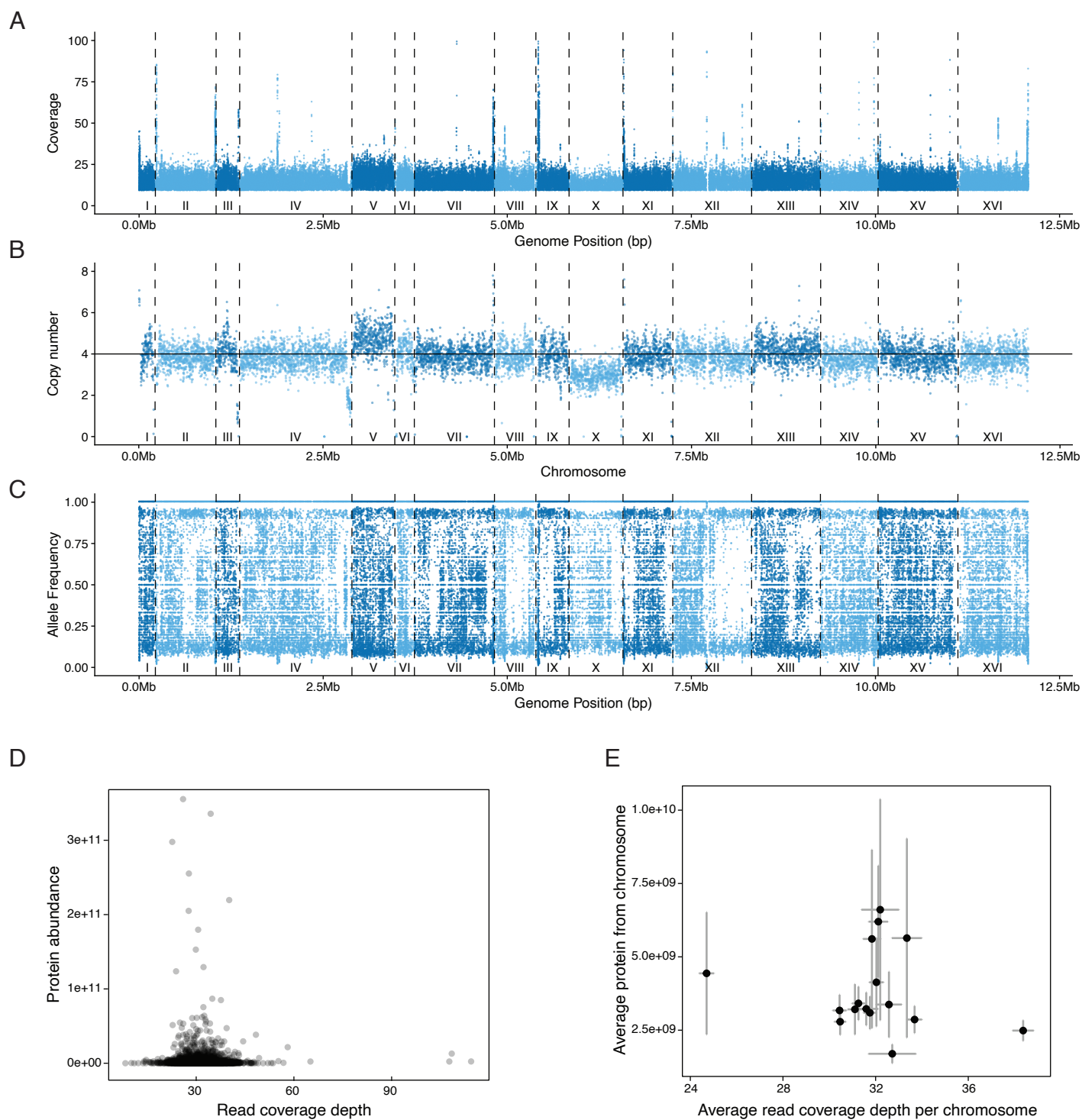

**Supplementary Figure 1**

Supplement: jkad293_Supplementary_Data [file jkad293_supplementary_data.zip › Supplementary_Figure_1_G3-2023-404592.pdf]

A

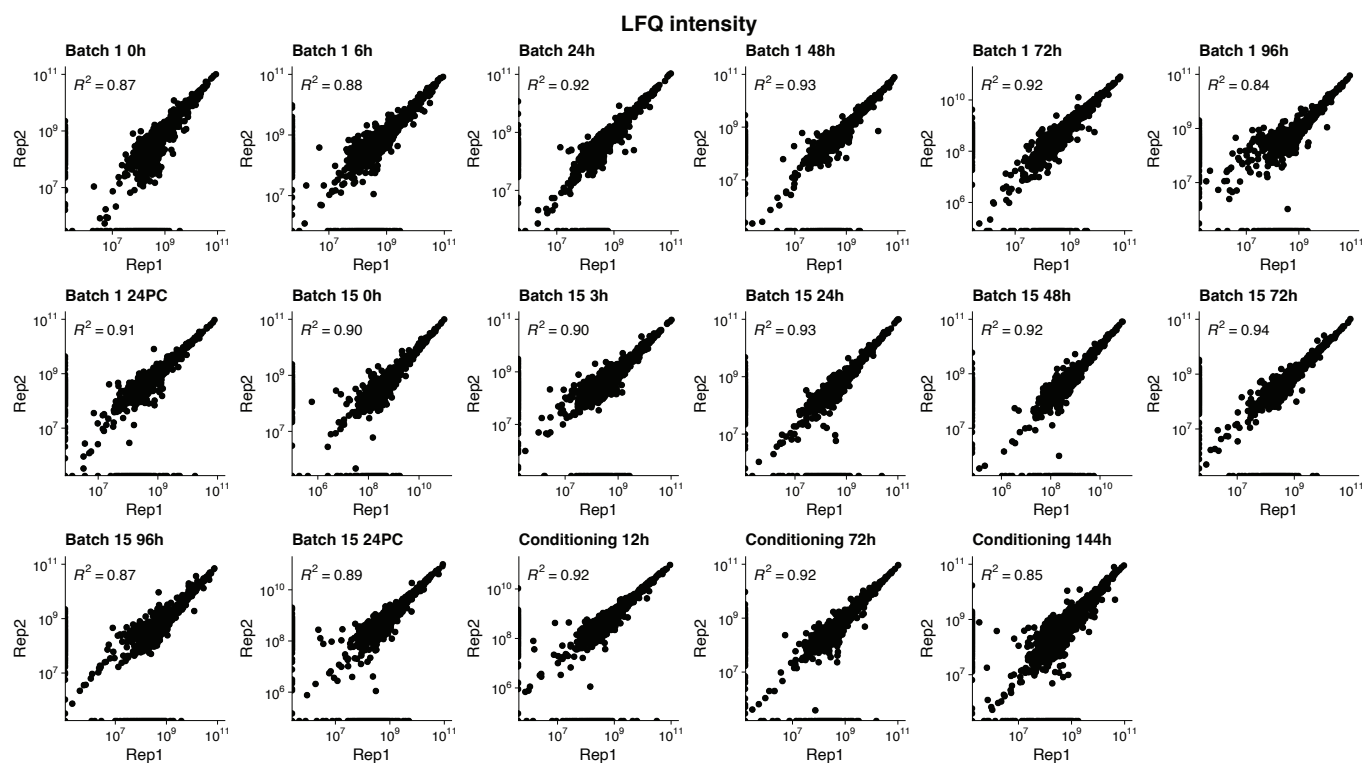

B

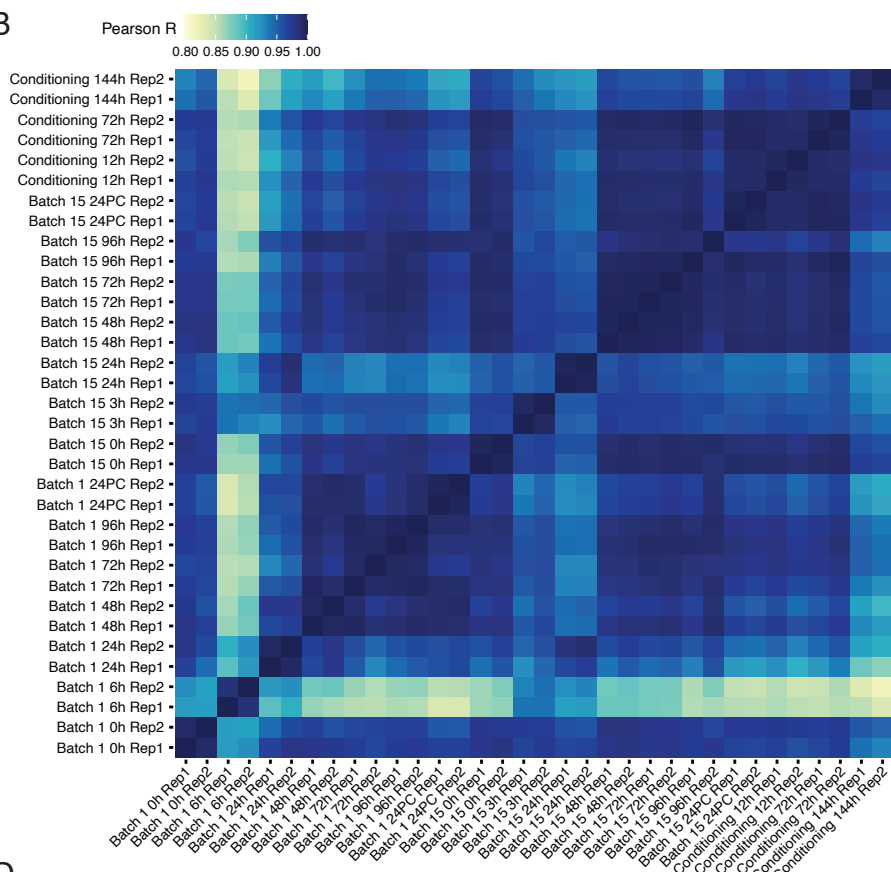

C

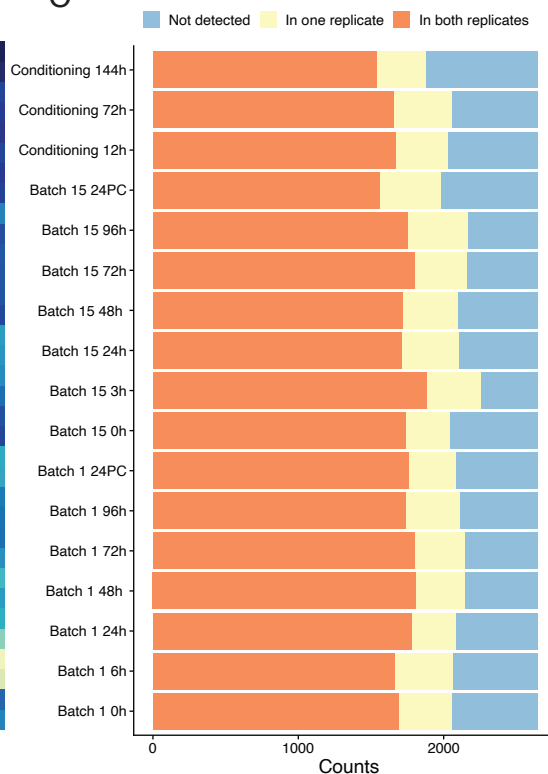

D

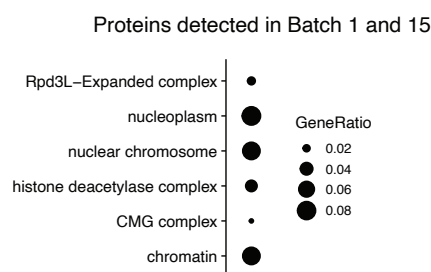

E

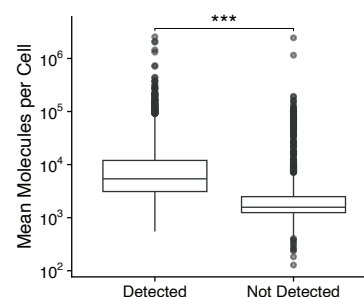

**Supplementary Figure 2**

Supplement: jkad293_Supplementary_Data [file jkad293_supplementary_data.zip › Supplementary_Figure_2_G3-2023-404592.pdf]

A

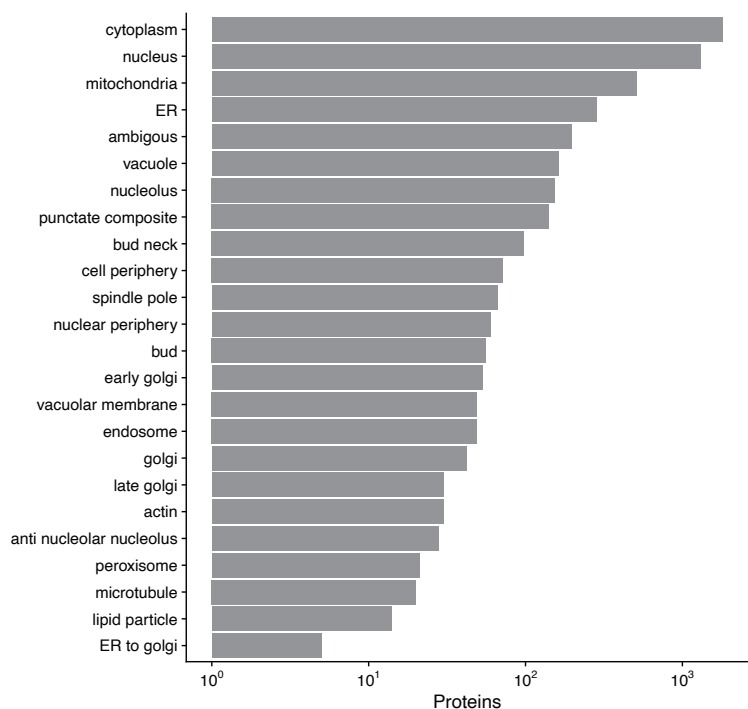

B

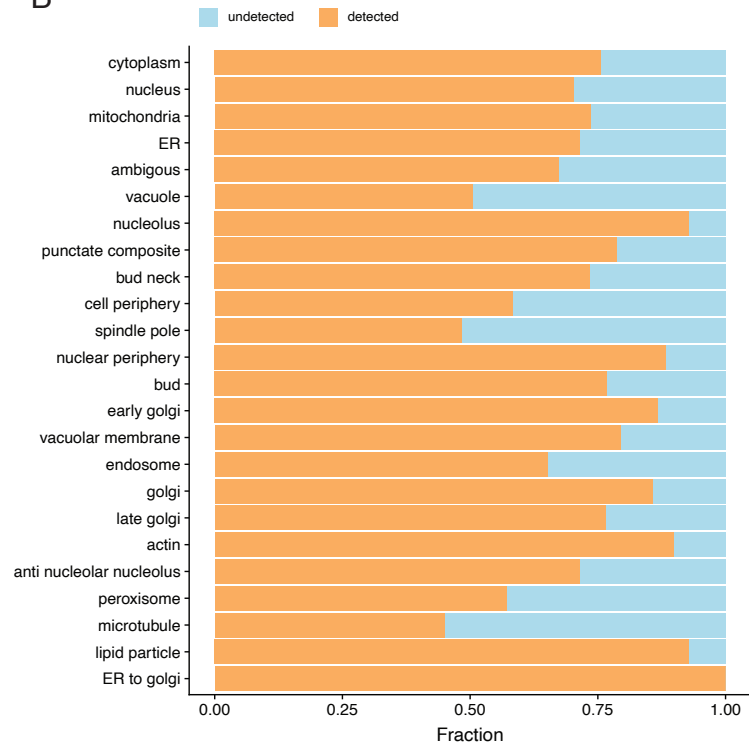

C

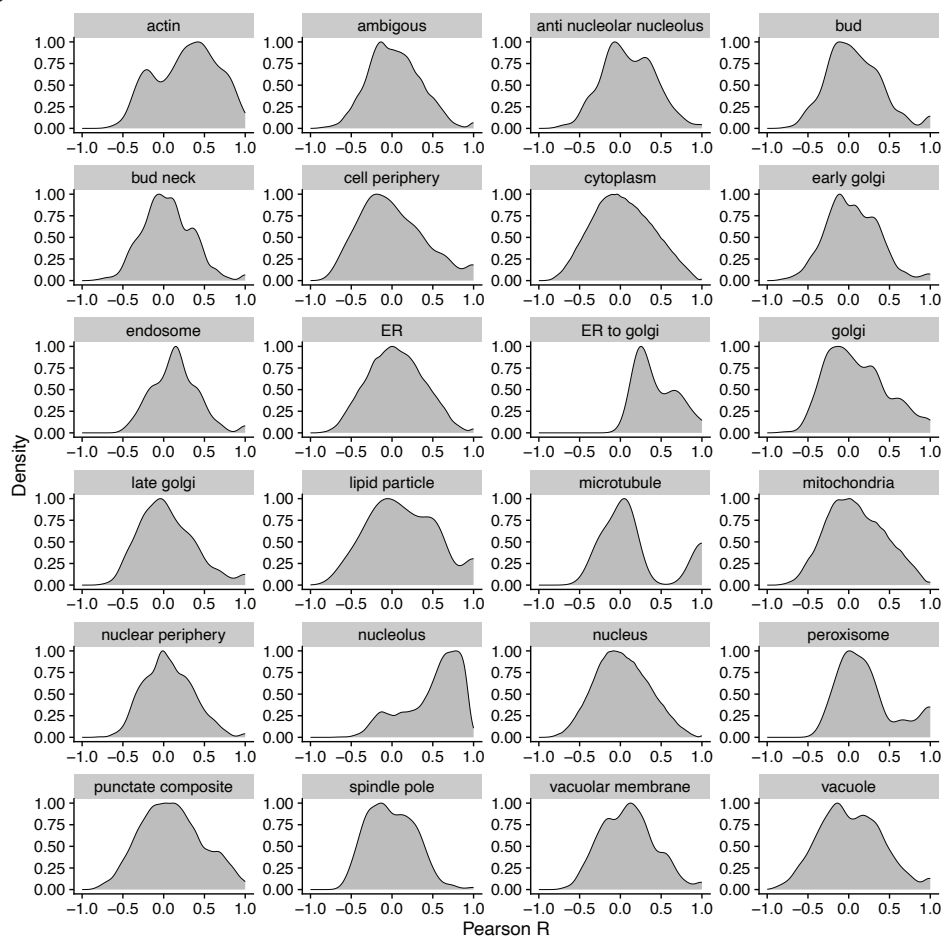

D

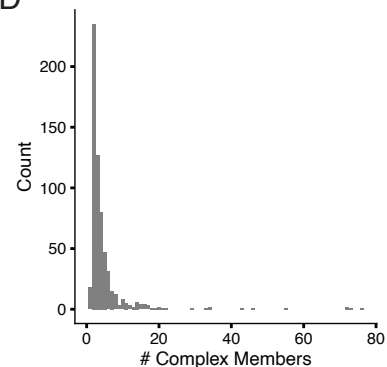

E

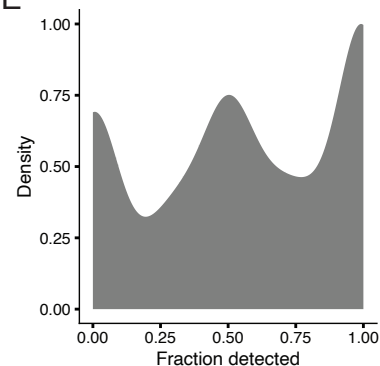

Supplementary Figure 3

Supplement: jkad293_Supplementary_Data [file jkad293_supplementary_data.zip › Supplementary_Figure_3_G3-2023-404592.pdf]

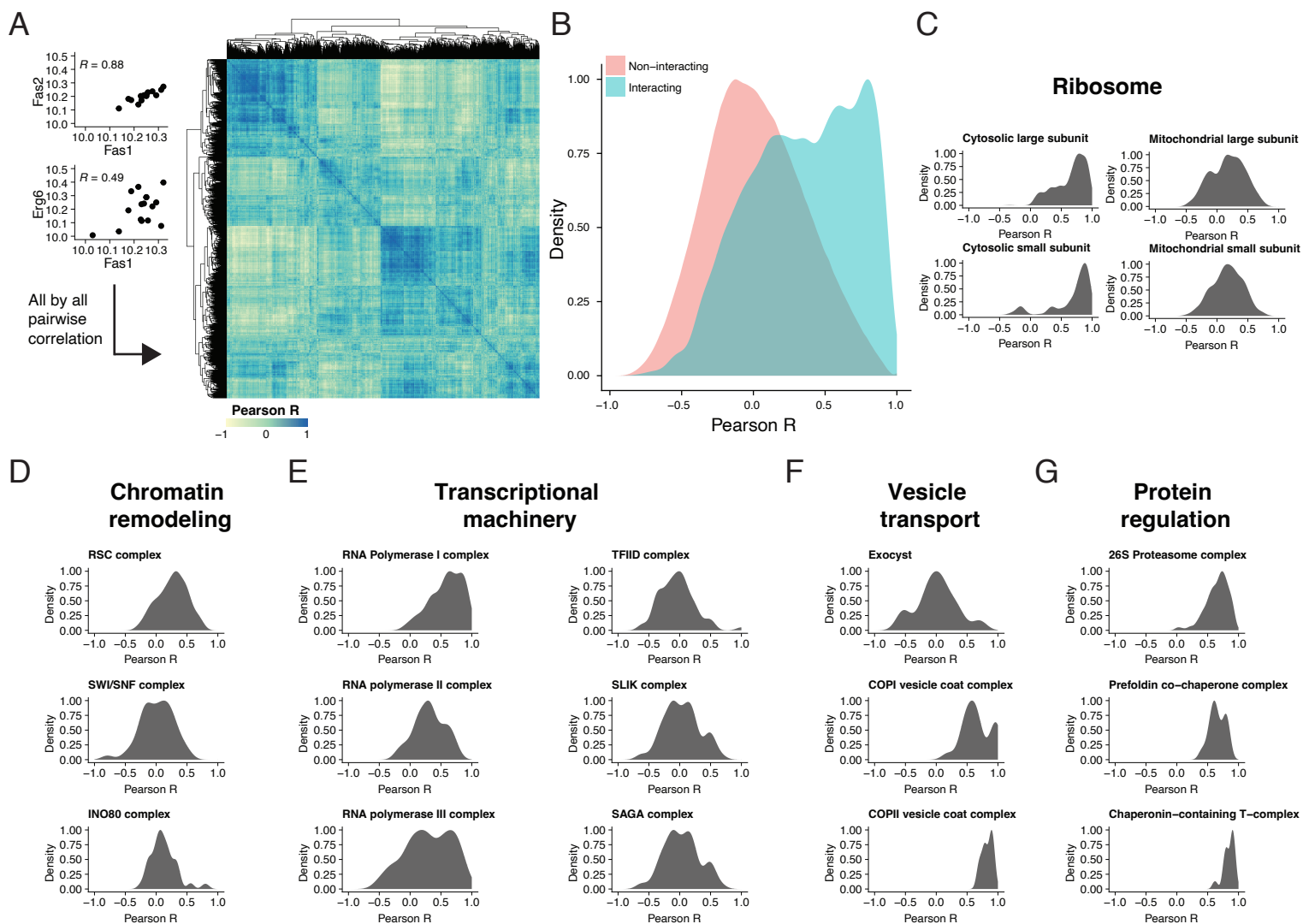

**Supplementary Figure 4**

Supplement: jkad293_Supplementary_Data [file jkad293_supplementary_data.zip › Supplementary_Figure_4_G3-2023-404592.pdf]

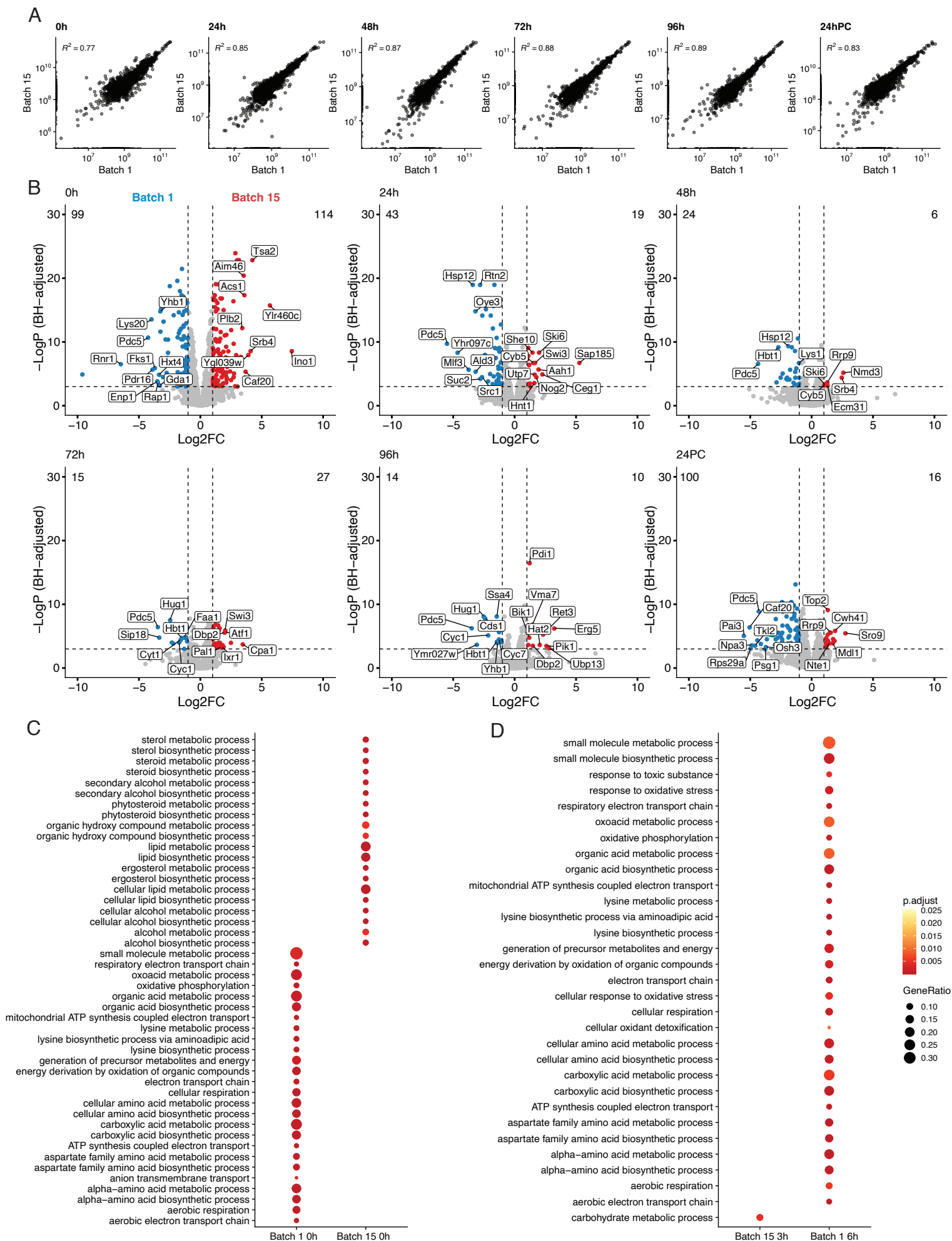

Supplement: jkad293_Supplementary_Data [file jkad293_supplementary_data.zip › Supplementary_Figure_5_G3-2023-404592.pdf]
